# Channel network structure determines genetic connectivity of landward–seaward *Avicennia marina* populations in a tropical bay

**DOI:** 10.1002/ece3.6829

**Published:** 2020-10-16

**Authors:** Ludwig Triest, Tom Van der Stocken, Abbie Allela Akinyi, Tim Sierens, James Kairo, Nico Koedam

**Affiliations:** ^1^ Research Group Plant Biology and Nature Management Vrije Universiteit Brussel Brussels Belgium; ^2^ Department of Oceanography and Hydrography Kenya Marine and Fisheries Research Institute Mombasa Kenya

**Keywords:** *Avicennia*, gene flow models, genetic structure, mangrove, microsatellites

## Abstract

Mangrove ecosystems along the East African coast are often characterized by a disjunct zonation pattern of seaward and landward *Avicennia marina* trees. This disjunct zonation may be maintained through different positions in the tidal frame, yielding different dispersal settings. The spatial configuration of the landscape and coastal processes such as tides and waves is expected to largely influence the extent of propagule transport and subsequent regeneration. We hypothesized that landward sites would keep a stronger genetic structure over short distances in comparison with enhanced gene flow among regularly flooded seaward fringes. We tested this hypothesis from densely vegetated *A. marina* transects of a well‐documented mangrove system (Gazi Bay, Kenya) and estimated local gene flow and kinship‐based fine‐scale genetic structure. Ten polymorphic microsatellite markers in 457 *A. marina* trees revealed no overall significant difference in levels of allele or gene diversities between sites that differ in hydrological proximity. Genetic structure and connectivity of *A. marina* populations however indicated an overall effect of geographic distance and revealed a pronounced distinction between channels and topographic setting. Migration models allowed to infer gene flow directionality among channels, and indicated a bidirectional steppingstone between seaward and nearest located landward stands. Admixed gene pools without any fine‐scale structure were found within the wider and more exposed Kidogoweni channel, suggesting open systems. Elevated kinship values and structure over 5 to 20 m distance were only detected in two distant landward and seaward transects near the mouth of the Mkurumuji River, indicating local retention and establishment. Overall, our findings show that patterns of *A. marina* connectivity are explained by hydrological proximity, channel network structure, and hydrokinetic energy, rather than just their positioning as disjunct landward or seaward zones.

## Graphical abstract

Using ten polymorphic microsatellite markers in 457 *A. marina* trees, we show that connectivity patterns of landward–seaward *A. marina* populations in Gazi Bay (Kenya) are explained by hydrological proximity, channel network structure, and hydrokinetic energy, rather than just their positioning as disjunct landward or seaward zones. Genetic structure and connectivity of *A. marina* populations indicated an overall effect of geographic distance and revealed a pronounced distinction between channels and topographic setting.

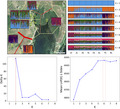

## 1 INTRODUCTION

Mangroves represent structurally and functionally characteristic forests, predominantly along tropical and subtropical coastlines and mostly occupying sheltered (low‐gradient) tidal flats in estuaries, deltas, and lagoons. Exposed to the dynamic conditions of these intertidal environments (e.g., tidal flooding, salinity fluctuations), mangrove trees and shrubs display a number of adaptive strategies such as salt‐secreting glands, aerial roots, and the production of hydrochorous propagules (i.e., dispersal units) (Tomlinson, 2016). Transport of these propagules allows for gene flow within and between mangrove populations, and determines the ability of species to track climate‐driven changes in the spatial distribution of suitable habitat. Estimates of gene flow and knowledge on the factors that determine the distribution of genetic diversity is not only of theoretical interest, but can be useful to inform management and conservation of these coastal ecosystems (Balbar & Metaxas, 2019; Carr et al., 2017; Nakajima et al., 2017; Pujolar et al., 2013; Schwarzbach & Ricklefs, 2001).

Mangrove propagules are transported by the hydrokinetic energy from waves, rivers, tides, near‐shore and open‐ocean currents, as well as wind energy, over short (near the parent tree) to transoceanic distances. Dispersal potential and the patterns of gene flow depend on the cumulative effect of a wide range of factors, such as propagule buoyancy and viability period, species‐specific propagule morphological traits (size and shape), landscape complexity, and the position of the parent tree in the tidal frame (Van der Stocken et al., 2019). Rabinowitz (1978) proposed that the interacting effects of water depth with species‐specific propagule traits (“tidal sorting”) might explain the differential distribution (zonation) of mangrove species along the tidal gradient. Similarly, the position of populations relative to open‐water channels and the spatial arrangement of these channels and their tidal currents may determine rates of hydrological connectivity and influence population genetic structure (Hughes et al., 2009; Pilger et al., 2017; Sander et al., 2018; Thomaz et al., 2016). However, while “riverscape genetics” is an active field of research and the importance of tidal inundation, dispersal traits, and establishment in determining mangrove forest structure (intertidal zonation) has been studied extensively (e.g., Clarke et al., 2001; Jiménez & Sauter, 1991; Rabinowitz, 1978; Sousa et al., 2007; Wang et al., 2019), few studies have linked these factors to the local (<10 km) and fine‐scale (i.e., within‐population) spatial genetic structure of mangroves.

Previous studies found correlations between genetic differentiation and geographic distance (e.g., Binks et al., 2018; Cerón‐Souza et al., 2015; Mori et al., 2015), a correlation known as "isolation by distance" (Rousset, 1997; Wright, 1943). However, aspects such as founding history, variations in dispersal traits, and interactions with spatially heterogeneous landscapes (i.e., spatial variation in transport resistance) have challenged the explanatory power of this model (e.g., Dodd et al., 2002; Maguire, Saenger, et al., 2000; Millán‐Aguillar et al., 2016; Wee et al., 2014). As a result, alternative hypotheses have been proposed that account for dispersal limitation (isolation‐by‐dispersal limitation; Orsini et al., 2013), the effect of ecological and geographical barriers (isolation‐by‐barrier; Ricketts, 2001), or incorporate resistance surfaces that reflect landscape properties (“roughness”) (isolation by resistance; McRae, 2006). For example, fine‐scale spatial genetic structure was observed in two *Avicennia* species along the Brazilian coast and explained by restricted pollen and propagule dispersal (Mori et al., 2015). Cisneros‐de la Cruz et al. (2018) focused on two physiognomic types (tall and scrub) of *Rhizophora mangle* L. (Rhizophoraceae) in the Yucatan Peninsula, and found comparable genetic differences within populations from the same site as between populations from different sites. The authors ascribed this pattern to high autogamy rates, asynchronous phenology between populations, as well as limited dispersal due to the interaction between large propagule size and the physical barrier presented by the species’ intricate root system. Similarly, Ngeve et al. (2017) mentioned the role of propagule retention in explaining the fine‐scale spatial genetic structure of *Rhizophora racemosa* G. Mey. (Rhizophoraceae) in a Cameroonian estuary complex. However, fine‐scale spatial genetic structure was not observed in all of the estuaries studied, and where absent, was explained by the recent recolonization of areas that are cleared for coastal development (Ngeve, Van der Stocken, Menemenlis, et al., 2017). While these studies help clarifying the role of propagule (dispersal) traits and interactions with the spatial complexity of the landscape, only a handful of studies considered the role of intertidal position and local hydrological system. Based on a preliminary genetic analysis, Dahdouh‐Guebas et al. (2004) found few allele frequency differences between landward and seaward *Avicennia marina* (Forsk.) Vierh. (Acanthaceae) zones in a Kenyan mangrove forest, indicating that there might be less genetic interchange between these intertidal zones than within each zone. Recently, Chablé Iuit et al. (2020) studied the genetic diversity and structure of *R. mangle* in the southern part of Quintana Roo (Mexico) and reported that the fine‐scale spatial genetic structure reflects contemporary processes such as restricted propagule dispersal and local hydrology.

The goal of this study is to characterize the genetic structure and diversity of the mangrove species *A. marina* in a coastal bay in Kenya, focusing on the effect of intertidal position and the structure of the area's channel network. More specifically, we aim to (a) analyze the genetic structure between seaward and landward mangrove patches, positioned along a same transect perpendicular to a channel; (b) estimate patterns of connectivity in the light of channel network structure; and (c) examine the fine‐scale spatial genetic structure of mangrove patches located along the same channel. The local‐ and fine‐scale spatial genetic structure of *A. marina* can be hypothesized to maintain higher levels of connectivity among the more regularly flooded seaward sites and higher kinship values (relatedness) with a stronger structure over short distances in higher intertidal (landward) sites. We test this hypothesis of confinement in landward sites versus open connectivity between seaward sites using densely vegetated transects in a well‐documented mangrove area (Gazi Bay, Kenya). The study site and species present an ideal case to undertake this study, given the disjunct (landward–seaward) pattern of *A. marina* in the area and the regional setting that is characterized by a series of open‐water channels. Throughout this manuscript, we use the term “landward” to refer to “higher intertidal” and “seaward” for “lower intertidal.” It is important to note that since channels have different orientations relative to the coastline, seaward does not necessarily mean oriented toward the sea. Instead, the terms “landward” and “seaward” reflect different geographical proximities (distant vs. close, respectively) to one of the three major water channels that cross the area's mangrove forest.

## 2 MATERIALS AND METHODS

### 2.1 Study area

**FIGURE 1:**
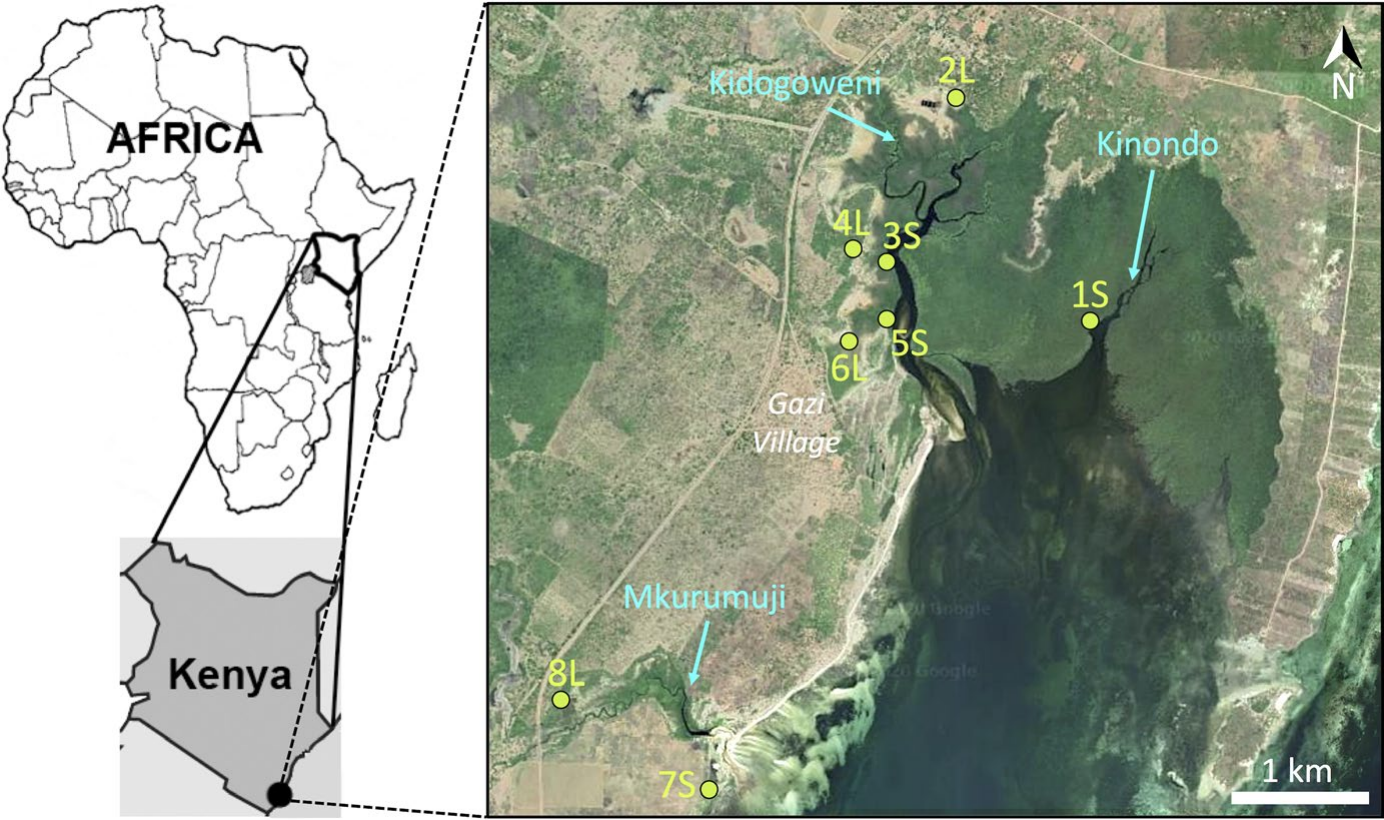
Map of Gazi Bay mangrove area and position of eight *Avicennia marina* seaward and landward sites. Map data: Google Earth, CNES/Airbus, Maxar Technologies

For the purpose of this study, data were collected in a mangrove forest in Gazi Bay (4°26′S, 39°30′E), about 45 km south–southwest of Mombasa (Figure 1). Climate conditions in the region are influenced by monsoon winds, with long rains during the southeast monsoon (March–July) and short rains during the northeast monsoon (November–December) (Kitheka et al., 1996). Gazi Bay consists of a shallow tropical water system characterized by a mangrove forest that covers >600 ha (Hemminga et al., 1994). The forest is dominated by *Avicennia marina* (Forsk.) Vierh. (Acanthaceae), *Sonneratia alba* J. Smith (Lythraceae), *Rhizophora mucronata* Lam. (Rhizophoraceae), *Ceriops tagal* (Perr.) C.B. Robinson (Rhizophoraceae), *Bruguiera gymnorrhiza* (L.) Lam. (Rhizophoraceae), and *Xylocarpus granatum* J. Koenig (Meliaceae) (Gallin et al., 1989), of which the former four are most abundant (Neukermans et al., 2008). The area's hydrological network is characterized by three major channels: Kinondo, Kidogoweni, and Mkurumuji (Figure 1), crossing the mangrove forest in the eastern, central, and southwestern part of the bay, respectively. In contrast to the Kinondo tidal creek, which lacks direct riverine input, the Kidogoweni river estuary receives surface freshwater input from the Kidogoweni River in the northern part of the bay (Kitheka, 1997). River discharge has seasonal variation, peaks in the wet season, and is higher for the Mkurumuji River than for the Kidogoweni River (Kitheka et al., 1996). Due to the riverine input, salinity in the Kidogoweni river estuary varies greatly, from 2 to 38 PSU, while salinity in the Kinondo tidal creek fluctuates between 22 and 38 PSU, and with salinity maxima (38 PSU) found in the upper parts of these channels during the dry season (Kitheka, 1997). Previous studies in the growth zone of *A. marina* revealed that salinity can also fluctuate strongly over the course of a tidal cycle (Tonné et al., 2017) and seasonally (Robert et al., 2014). Besides riverine influence, the water circulation in the area is controlled predominantly by the strong semi‐diurnal tides that enter the bay via a ca. 3.5‐km‐wide entrance (to the Indian Ocean) in the south (Kruyt & van den Berg, 1993), with a spring tide range of 3.2 m and neap tide range of 1.4 m (Kitheka, 1997). These tides cause strong and reversing currents that are characterized by relatively stronger ebb than flood currents (tidal asymmetry), allowing for net export (Kitheka et al., 1996).

### 2.2 Study species

*Avicennia marina* is the most widely distributed of all mangrove species, found across the Indo‐Pacific, between latitudes 25°N and 38°S. It has been shown that *A. marina* is able to grow and reproduce across a relatively broad range of climatic, saline, and tidal conditions (Duke et al., 1998). Salt‐excreting glands in its leaves allow the species to better tolerate high salinities compared to other mangrove species (Clough, 1984). In a sedimentation experiment in Gazi Bay, Okello et al. (2014) showed that *A. marina* trees respond, and may adapt, relatively rapid to high sedimentation events. In addition, *A. marina* trees generally show high fecundity, with propagule counts between 422 and 5,210 propagules annually per tree (for stands in southeastern Australia; Clarke, 1992). The combination of these factors may help explain the wide occurrence of this species. Across its range, effective population size and genetic diversity are highest in core populations and decrease near the species’ range edges (De Ryck et al., 2016).

In our study region, *A. marina* shows a disjunct (landward–seaward) distribution pattern across the intertidal zone, typically separated by formations of *C. tagal* and *R. mucronata*, with the trees from both zones often showing apparent physiognomic differences: trees in the landward fringe can show a rather shrub‐like appearance with an average height of ca. 3 m, and taller and more robust trees with an average height of ca. 10 m in the seaward fringe (Dahdouh‐Guebas et al., 2004). While the seaward stands usually are close canopy forests with dense pneumatophore structures, the landward stands are open with interspersed sand flats. Phenological research in the study area revealed that propagule fall peaks during the wet season (April–May; Wang’ondu et al., 2010). Shade intolerance and high predation rates on its propagules are believed to limit the distribution of *A. marina* across the intertidal zone (Smith III, 1987).

The propagules of *A. marina* consist of a single embryo surrounded by a thin pericarp (Tomlinson, 2016). Reported flotation and viability times for *A. marina* propagules are relatively short, spanning a couple of days to weeks (Clarke et al., 2001; Clarke & Myerscough, 1991; Steinke, 1986). However, it should be mentioned that the duration of the experimental trials on which these findings are based may be too short to obtain meaningful frequency distributions of these propagule traits, and should ideally extend beyond maximum values (Van der Stocken et al., 2019). Floating periods in other *Avicennia* species exceed several months (Alleman & Hester, 2011; Rabinowitz, 1978; Van der Stocken et al., 2018), and previous studies reported that the buoyancy of *A. marina* propagules varies greatly among estuaries (Steinke, 1986). Propagules are rather compact compared to the propagules from other mangrove species, potentially reducing steric hindrance by physical structures such as aerial roots (Van der Stocken et al., 2015).

### 2.3 Sample collection

A total of 457 *A. marina* individual trees were sampled during July 2017 in eight locations (Table 1) of which the geographical distribution (Figure 1) allows to assess the goals of this study. Sampling locations with different hydrological proximity were chosen at different positions in the tidal frame, along main channels and side channels, and at nonchannel connected sites. These consist of seaward sites along Kinondo (1S), Kidogoweni (3S and 5S), and Mkurumuji (7S), and landward sites nearby Kidogoweni (2L, 4L, and 6L) and Mkurumuji (8L). The eight transects were each approximately 20 m wide and 100 m in length, and the number of sampled trees ranged from 53 to 61 per transect. GPS coordinates at the starting point of each transect were taken. The distance between each subsequent sample within the densely vegetated transects varied between 2 and 5 m such that a suite of neighboring trees was included. Most of the trees were adult (3–5 m height), only few young established trees (2–5 years) were sampled. We discarded patches of seedlings or juveniles to avoid any effect of sibling dominance on the kinship values. Two bright green leaves were collected per individual, dried in open air, and preserved in paper envelopes with silica gel for transportation and handling within 1 month.

**TABLE 1:** Location details of seaward (S) and landward (L) *Avicennia marina* sites of the Gazi Bay mangrove area (Kenya)

Site	Location	Latitude	Longitude
1S	Kinondo, seaward	−4,417,528	39,524,250
2L	Kidogoweni, landward	−4,402,889	39,515,194
3S	Kidogoweni, seaward	−4,413,819	39,510,814
4L	Kidogoweni, landward	−4,412,842	39,508,556
5S	Kidogoweni, seaward	−4,417,442	39,510,903
6L	Kidogoweni, landward	−4,419,033	39,508,186
7S	Mkurumuji, seaward	−4,448,769	39,499,150
8L	Mkurumuji, landward	−4,443,028	39,489,250

### 2.4 DNA extraction and microsatellite primers

Genomic DNA was extracted from approximately 20 mg of dried leaf tissue using the E.Z.N.A. SP plant DNA Mini kit (Omega bio‐tek, Norcross, GA, USA). A multiplex polymerase chain reaction (PCR) consisted of in total 10 microsatellite markers (Appendix S1). Six of the markers were previously developed by Maguire, Edwards, et al. (2000) and Geng et al. (2007) for *A. marina*. To ensure high resolution of genotyped individuals, we developed four new primers for polymorphic microsatellite markers using source material from Gazi Bay. For the development of these new markers, an Illumina paired‐end library was constructed and sequenced using the Illumina HiSeq platform at Macrogen (Seoul, Republic of Korea). SSR_pipeline (Miller et al., 2013) was used to find microsatellites. Out of 19.3 million 100 bp paired‐end reads, 1.4 million pairs were successfully joined by the module joinseqs. The module SSR_search found 5,178 dinucleotide SSRs with at least 10 repeats, 362 trinucleotide SSRs with at least 8 repeats, and 227 tetranucleotide SSRs with at least 6 repeats. We used Batchrimer3 (You et al., 2008) to design primers and 56 primer pairs were selected for synthesis on the basis of number of repeats and expected fragment length. Using Multiplex Manager (Holleley & Geerts, 2009), we added 4 new polymorphic loci to the previously existing multiplex to form one single multiplex reaction of 10 amplifiable primer pairs. Primers were fluorescence‐labeled with 4 different dye labels (6FAM/VIC/NED/PET), and a primer mix was made by mixing 0.2 µM of each primer together. Multiplex PCRs consisted of 6.25 µl master mix (Qiagen Multiplex PCR kit), 1.25 µl primer mix, 2.5 µl H_2_O, and 2.5 µl of genomic DNA. PCR was performed in a thermal cycler (Bio‐Rad MyCycler) with the following conditions: an initial denaturation of 95°C for 15 min followed by 35 cycles of: 30 s denaturation at 95°C, 90 s annealing at 57°C and 80 s elongation at 72°C followed by a final extension of 30 min at 60°C. PCR products were separated on an ABI3730XL sequencer (Macrogen, Seoul, Korea), and allele sizes were determined with GeneMarker V2.60 (SoftGenetics LLC, State College, USA).

### 2.5 Genetic analyses

Prior to population and individual‐based data analysis, we tested for genotypic disequilibrium, potential null alleles, and overall resolution of the selected ten microsatellite markers in *A. marina*. A linkage test between all pairs of loci (1,000 permutations) gave no genotypic disequilibrium at the 0.05 level using FSTAT (v.2.9.3) (Goudet, 2001). No scoring errors, large allele dropouts, or null alleles were indicated using MICRO‐CHECKER (Van Oosterhout et al., 2004). The probability of identity (*PI*), namely whether two individuals could share an identical multilocus genotype by chance using GenAlEx (v.6.5; Peakall & Smouse, 2012), gave a cumulative *PI* for all polymorphic loci in each site of 1.4 10^–5^–9.5 × 10^–6^, thereby providing ample resolution, even for siblings, potentially present in our subsequent sampling design that reached a *PI* of 1.8–7.2 × 10^–3^ (Appendix S2).

Basic population genetic variables were measured for each site: total number of alleles (*A*), mean number of alleles (*A*_M_), effective number of alleles (*A*_E_), allelic richness (*A*_R_) for 46 diploid samples, observed heterozygosity (*H*_O_), unbiased expected heterozygosity (*H*_E_), and population inbreeding coefficient (*F*_IS_) using FSTAT and GenAlEx. The genetic structure among sites (*F*_ST_), inbreeding within sites (*F*_IS_), and overall inbreeding (*F*_IT_) was calculated via AMOVA–*F*_ST_ at 999 random permutations using GenAlEx v.6.5, thereby allowing to estimate overall connectivity levels as *N*m = *F*_ST_/(1‐4*F*_ST_) under the assumption of an island migration model within the Gazi Bay. An additional hierarchical AMOVA was performed and *F*‐statistics were calculated, considering three channels (Kinondo, Kidogoweni, and Mkurumuji) as regions, and using 999 random permutations. Pairwise genetic differentiation (*F*_ST_) was used to produce a PCoA at population level and together with a pairwise geographic Euclidean distance to perform a Mantel test using 1,000 permutations in GenAlEx (v.6.5). Pairwise genotypic differentiation was used to produce a PCoA at individual level. The overall *F*_IJ_ kinship coefficient (Loiselle et al., 1995) for all sites of *A. marina* in Gazi Bay was estimated for five mean distance classes at 0.27, 0.44, 1.12, 1.78, and 4.2 km, as were automatically generated when requesting an equal number of pairwise comparisons within each class by SPAGeDi 1.5a (Hardy & Vekemans, 2002) and using the whole sample as a reference. These distance classes represent threshold values as indicated by a Mantel test. Two zonation groups (seaward and landward) and two age groups (young and mixed older) were tested for differences in their *A*_R_, *H*_O_, *H*_E_, *F*_IS,_ and *F*_ST_ using 1,000 permutations in FSTAT. The *F*_IJ_ kinship coefficient was estimated between reciprocal pairs of seaward and landward sites using SPAGeDi. An assignment of individuals to their “self” population or to another population was done with the “leave‐one‐out” option in GenAlEx.

A Bayesian clustering analysis at individual level was carried out in STRUCTURE version 2.3.4 (Pritchard et al., 2000) using an admixture model with correlated allele frequencies. The model ran 20 iterations for each *K* value from 1 to 8; the burn‐in period was 100,000 with 500,000 Markov chain Monte Carlo (MCMC) repeats. The optimal *K* value was inferred with the Δ*K* statistic (Evanno et al., 2005), from LnP(*K*), and the Puechmaille (2016) method using Structure Harvester (Earl & von Holdt, 2012) and CLUMPAK (Kopelman et al., 2015), calculated with StructureSelector (Li & Liu, 2018). The software BARRIER 2.2 (Manni et al., 2004) was used to detect the location of sharp genetic changes between neighboring populations based on one overall pairwise *F*_ST_ matrix and 10 pairwise *F*_ST_ matrices of every microsatellite locus, allowing a maximum of one barrier per matrix. Even though bootstrapped matrices are commonly performed when only a single differentiation matrix is available (e.g., from sequences), we opted to calculate from superposition of basic data from different *F*_ST_ matrices at locus level. The thickness of barrier lines thus will be based on the additivity of matrices accounting for the variability of different markers that we consider as a preferred informative and valid method over bootstrapping a single mean *F*_ST_ matrix.

MIGRATE‐*n* (Beerli, 2006; Beerli & Palczewski, 2010) was used to estimate the mutation‐scaled population sizes (Theta) and immigration rates (M). We considered 2 migration scenarios at different spatial scales: (A) along a landward‐seaward distribution near the Kidogoweni channel, and (B) between the tree major channels across the bay. Uni‐ and bidirectional recent historical migration/expansion models were tested. Specific hypotheses testing on directionality were considered in panmixia, source–sink, and steppingstone models for (A) the migration between seaward sites (S3, S5) and landward sites (L4, L6) of disjunct vegetation zones in close vicinity and along the same channel (Kidogoweni), and for (B) the migration between the three channels within the Gazi Bay area where we considered the most seaward sites of each channel mouth (S1, S5, and S7). The Brownian model was tested locus by locus along with the product of all distributions of all loci and was balanced for a subsample of 20 individuals in each site. Uniform prior distribution settings (min, max, delta) were Theta = 0.0, 10.0, 0.1 and *M* = 0.0, 100, 10.0. The number of recorded steps was 10^6^ at a sampling frequency of 10^3^ after an initial burn‐in. Each run implemented the infinite allele model. Initial values were computed using *F*_ST_. The mutation rate was calculated from the data, following the above‐mentioned settings, computing two replicate chains (with different seed). We used the Bezier thermodynamic integration (Beerli & Palczewski, 2010) for calculating the Bayes factors from marginal likelihoods giving model probabilities. The effective number of immigrants per generation (*N* *e* *m*) was calculated as [Theta × M]/4 (Kennedy et al., 2016) for the best‐fit model of each scenario.

A fine‐scale spatial autocorrelation of individuals at transect level was performed with a kinship coefficient (*F*_IJ_) analysis (Loiselle et al., 1995) over five distance classes (0–5, 5–10, 10–25, 25–50, and 50–100 m) using SPAGeDi 1.5a and tested for significance with 1,000 permutations using each within‐category as a reference. The slope of the regression over the full distance of each transect (up to 99 m) was tested with 1,000 permutations. Within transects, each spanning ca. 100 m in length, distance classes were defined based on a first test considering an equal number of pairwise comparisons in five classes. Elevated kinship values were within less than 21 m. Therefore, we opted to use relevant distance classes (0–5, 5–10, 10–25, 25–50, and 50–100 m) that allowed differentiating within these shortest distances rather than beyond. The sampling strategy of 60 trees over 100 m × 20 m transects also allowed considering truly “neighbor” trees within the 5 m distance class. Furthermore, we calculated the *Sp*‐statistic, which is proposed as an informative parameter about survival strategy for diploids as *Sp* = −b_log_/(1−F_1_) (Vekemans & Hardy, 2004), where b_log_ is the slope of the ln regression and F_1_ represents the average kinship coefficient (*F*_IJ_) between neighboring individuals in the first distance class (0–5 m). Under an assumption of isolation‐by‐distance (valid in Gazi Bay) and two‐dimensional (i.e., 100 m × 20 m transects) space, the neighborhood size can be estimated as *Nb* = 1/*Sp* (Vekemans & Hardy, 2004).

## 3 RESULTS

### 3.1 Genetic diversity levels

In *A. marina* sites of Gazi Bay, the total number of alleles observed in the considered ten loci was 52 (34–42), with a mean number of alleles (*A*_M_) ranging between 3.5 and 4.2, an effective number of alleles (*A*_E_) between 2.0 and 2.5, and an adjusted allelic richness (*A*_R_) between 3.3 and 4.1 (Table 2). The overall observed heterozygosity (*H*_O_ = 0.480) was very similar to the expected heterozygosity (u*H*_E_ = 0.500). The within‐population inbreeding (mean *F_IS_* = 0.059) ranged from −0.014 to 0.147 and was significant only for site 5S (Table 2). A comparison of population genetic variables between two groups of seaward and landward transects revealed no significant (*p* > .05) differences in the levels of *A*_R_, *H*_O_, *H*_E,_ and *F*_IS_, whereas two young stands (2L and 7S) showed slightly higher allelic richness (*A*_R_ = 4.0 versus 3.5; *p* = .011) and gene diversity (*H*_E_ = 0.553 versus 0.483; *p* = .029) compared to eight older stands. Overall, we observed very similar amounts for basic population genetic variables for most *A. marina* sites within the bay.

**TABLE 2:** Population genetic variables of *Avicennia marina* sites in Gazi Bay, Kenya. N: number of genotyped samples; *A*: number of alleles; *A*_M_: mean number of alleles; *A*_E_: effective number of alleles; *A*_R_: allelic richness at *k* = 46 diploid individuals; *H*_O_: observed heterozygosity; *uH* **_E_**: unbiased expected gene diversity; *F*_IS_: within‐population inbreeding coefficient (with * at *p* < .05 significance level and *** at *p* < .001). Mean *F*_IS_ taken from AMOVA. Standard errors are provided between brackets

Site	*N*	*A*	*A*_R_	*A*_M_	*A*_E_	*H*_O_	*uH*_E_	*F*_IS_
1S	59	37	3.6	3.7 (0.5)	2.2 (0.3)	0.500 (0.066)	0.493 (0.058)	−0.014
2L	56	42	4.1	4.2 (0.5)	2.4 (0.3)	0.537 (0.071)	0.533 (0.058)	−0.008
3S	61	37	3.6	3.7 (0.6)	2.4 (0.4)	0.498 (0.065)	0.516 (0.066)	0.035
4L	53	35	3.5	3.5 (0.4)	2.3 (0.4)	0.456 (0.063)	0.492 (0.065)	0.074
5S	53	35	3.5	3.5 (0.4)	2.2 (0.3)	0.415 (0.045)	0.486 (0.059)	0.147*
6L	57	38	3.7	3.8 (0.6)	2.2 (0.3)	0.458 (0.076)	0.457 (0.076)	−0.001
7S	59	40	3.9	4.0 (0.5)	2.5 (0.3)	0.525 (0.037)	0.572 (0.034)	0.083
8L	59	34	3.3	4.0 (0.4)	2.0 (0.3)	0.448 (0.049)	0.453 (0.053)	0.011
Overall	457	52	4.2	–	–	–	–	–
Mean	57	37	3.7	3.7 (0.2)	2.3 (0.1)	0.480 (0.021)	0.500 (0.021)	0.059***

### 3.2 Differentiation between sites

**FIGURE 2:**
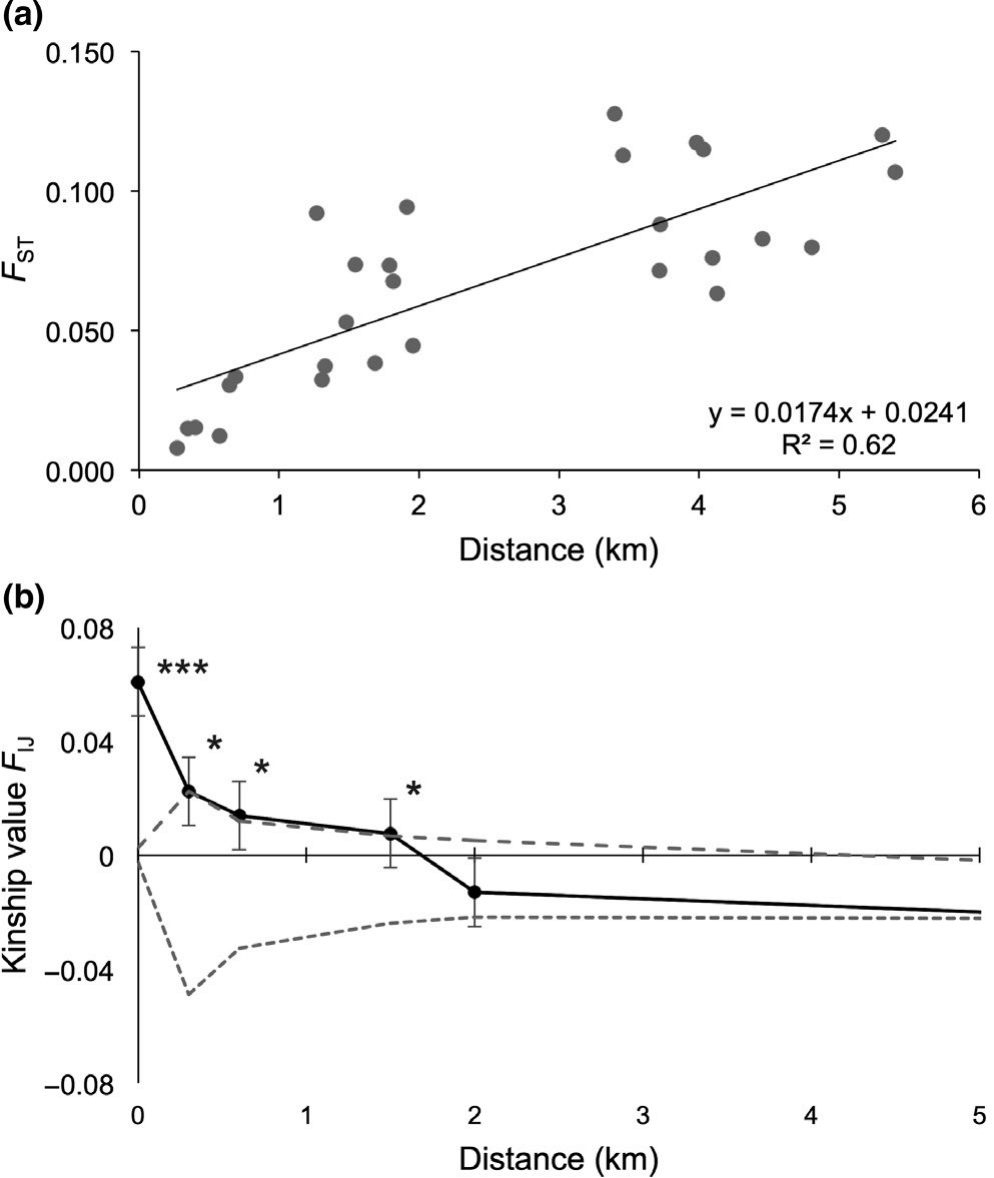
Isolation by distance of *Avicennia marina* sites within Gazi Bay with (a) positive regression of Mantel test over 5.4 km; (b) Spatial autocorrelation of *Avicennia marina* individuals across Gazi Bay showing decreasing kinship values (*F*_IJ_) from the within transect (zero km) to the among transect at 0.3, 0.6 km up to a maximum distance of 1.5 km (*** *p* < .001; * *p* < .05)

*Avicennia marina* within Gazi Bay showed an overall AMOVA–*F*_IT_ = 0.122, *F*_ST_ = 0.067, and *F*_IS_ = 0.059, though with all these low values at *p* = .001 (Table 3). Within the bay, 88% of *A. marina* genetic variation came from within individuals, whereas 7% was among the considered transects, giving an overall estimated gene flow of *Nm* = 3.5 (Table 3). A hierarchical AMOVA at the level of three channels showed *F*_RT_ = 0.043 (*p* < .001) and *F*_SR_ = 0.041 (*p* < .001) with as much variance among channels (4%) than among populations (4%). Pairwise differentiation ranged from 0.008 for transects in close vicinity (3S and 4L) to 0.126 for more distant landward sites (6L and 8L). Both PCoA at individual and population level showed a gradient along the first axis of the locations 8L, 7S, and 1S, although *A. marina* individuals clustered as a single cloud (Appendix S3). A comparison between the seaward and landward group at site level indicated no difference in their population differentiation *F*_ST_ (Table 4). However, at individual level, the estimated *F*_IJ_ kinship coefficients between reciprocal pairs of seaward and landward sites were close to zero and nonsignificant for all cases, indicating no traceable relatedness between disjunct zones. A Mantel test showed an isolation by distance (*y* = 0.017*x* + 0.024; *R* ^2^ = 0.62 at *p* = .003) over 5.4 km (Figure 2a) and the kinship value (*F*_IJ_) decreased significantly over the full distance (slope *b* = −0.03 at *p* < .001), with significantly (*p* < .05) higher kinship values at 0.3 km, 0.6 km, and up to a maximum distance of 1.5 km (Figure 2b).

**TABLE 3:** Summary of AMOVA and *F*‐statistics of *Avicennia marina* in Gazi Bay (Kenya), considering the populations and a hierarchical AMOVA at level of three channels

*Avicennia marina*	*df*	SS	MS	Est. Var.	%	*F*‐statistics	p‐value
Among Pops	7	164.461	23.494	0.182	7	*F*_ST_ = 0.067	0.001
Among Individual	449	1,204.062	2.682	0.148	5	*F*_IS_ = 0.059	0.001
Within Individual	457	1,090.000	2.385	2.385	88	*F*_IT_ = 0.122	0.001
Total	913	2,458.523		2.716	100	*N*m = 3.5	
Among Channels	2	89.076	44.538	0.117	4	*F*_RT_ = 0.043	0.001
Among Pops	5	75.385	15.077	0.110	4	*F*_SR_ = 0.041	0.001
Among Individual	449	1,204.062	2.682	0.148	5	*F*_ST_ = 0.082	0.001
Within Individual	457	1,090.000	2.385	2.385	86	*F*_IS_ = 0.059	0.001
Total	913	2,458.523		2.760	100	*F*_IT_ = 0.136	0.001

Abbreviations: %, percentage of total variation; *df*, degrees of freedom; Est. Var., estimated variance; MS, mean squares; SS, sum of squares.

**TABLE 4:** Pairwise comparisons of population genetic differentiation of *Avicennia marina* in Gazi Bay, Kenya. All pairwise *F*_ST_ values were low and significant either at *p* < .001 (***), *p* < .01 (**) or *p* < .05 (*)

	1S	2L	3S	4L	5S	6L	7S	8L
1S	–	***	***	***	***	***	***	***
2L	0.065	–	***	***	***	***	***	***
3S	0.074	0.034	–	*	**	***	***	***
4L	0.068	0.027	0.008	–	**	***	***	***
5S	0.054	0.032	0.016	0.014	–	**	***	***
6L	0.074	0.052	0.030	0.033	0.013	–	***	***
7S	0.084	0.087	0.078	0.065	0.074	0.113	–	***
8L	0.080	0.105	0.115	0.117	0.088	0.126	0.093	–

Colors indicate a gradient of low (green) to high (red) F_ST_ values

**FIGURE 3:**
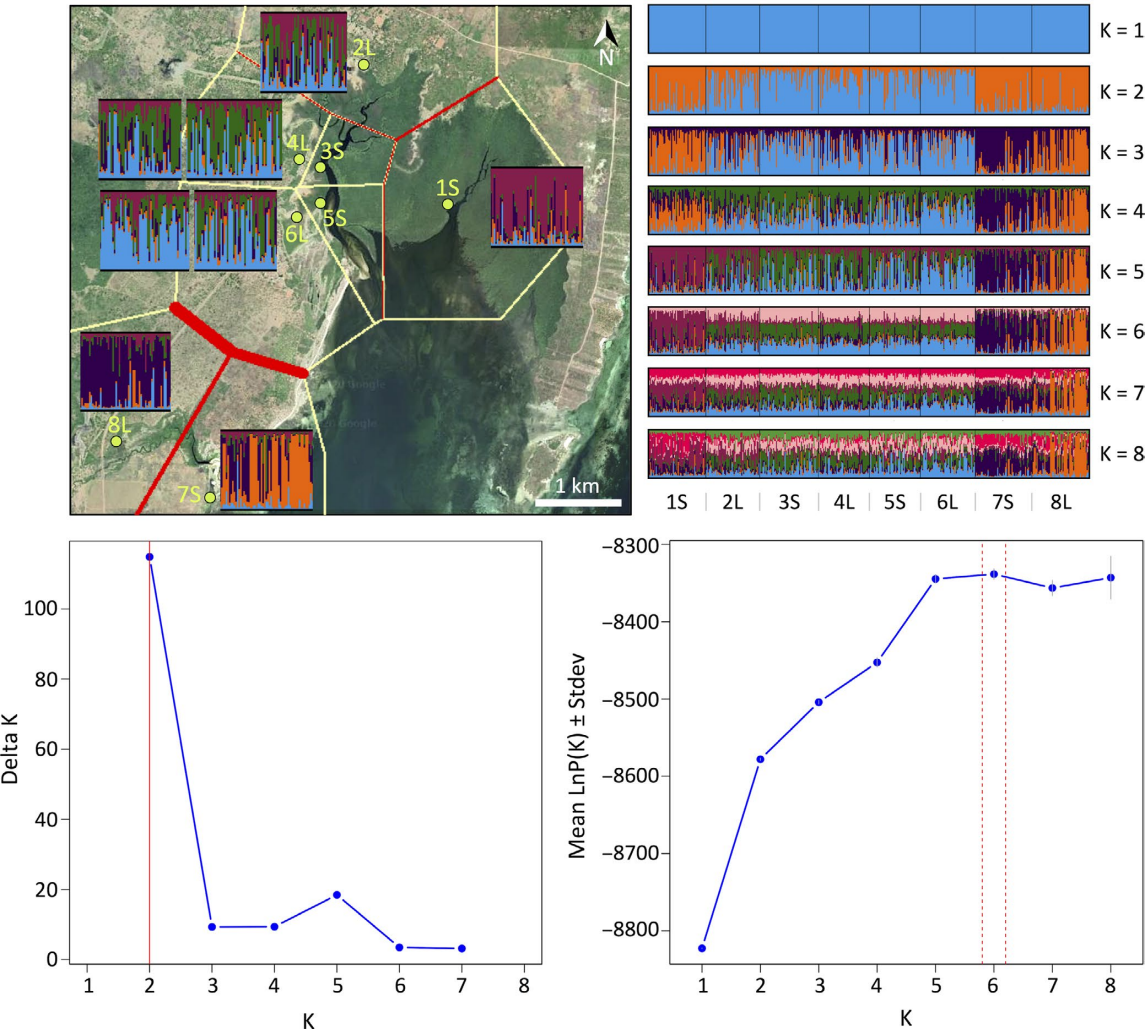
Map of Gazi Bay with results of Bayesian clustering analysis (STRUCTURE at *K* = 5) and with an overlay of first barriers (red lines) between neighboring sites (polygons in yellow). CLUMPAK bar diagrams are presented for 1 to 8 clusters as well as Delta K and LnP(K) graphs. Map data: Google Earth, CNES/Airbus, Maxar Technologies

A Bayesian clustering analysis of individual *A. marina* trees performed in STRUCTURE indicated a gradient of very admixed clusters (Figure 3). Delta K was highest for *K* = 2 (Delta *K* = 114) and reached a marginally higher value at *K* = 5 with Delta *K* = 18 (Figure 3). However, *K* = 5 showed best convergence with a mean LnP(*K*) = −8334 when compared to *K* = 2 (mean LnP(*K*) = −8578). *K* = 5 showed the highest mean similarity score (0.988) for CLUMPAK, and *K* = 5 was obtained with the Puechmaille (2016) method. Basically, Delta *K* of two clusters should be explained mainly from its proportionally large difference with *K* = 1 as a first step of calculating delta. Nonetheless, both *K* = 2 and *K* = 5 refer to a local substructure of the different channels 1S, 7S, and 8L versus a mixed group (Figure 3). However, both *K* = 2 and *K* = 5 must be regarded as an estimation for the Gazi Bay area with limited cases of assignment of an individual to but a single gene pool. The assignment of individuals resulted in 54% to the “self” population, with highest proportion of numbers for 1S, 7S, and 8L, which corresponds to the abovementioned gene pools obtained with STRUCTURE. A BARRIER analysis showed minor breaks between 1S and neighboring sites and a major break between the Mkurumuji locations (7S and 8L) and their neighbors, thereby separating the Gazi Bay transects according to the three major water channels (Figure 3).

MIGRATE was used to estimate the mutation‐scaled population sizes and immigration rates using the Brownian model. The specific testing on gene flow directionality between disjunct seaward and landward sites along the Kidogoweni channel gave best support for a bidirectional steppingstone model (Table 5a). Estimates of mean population size for the populations of this best‐fit model were Theta = 0.17–0.39 with mean migration *M* = 1.1–9.2 (Bayesian analysis posterior distribution made available in Appendix S4). We obtained highest estimated gene flow from 3S toward 4L (*N* *e* *m* = 0.80) and from 3S toward 5S (*N* *e* *m* = 0.64). Lowest gene flow estimates were found from landward toward seaward stands (*N* *e* *m* = 0.10 from 6L to 5S; *N* *e* *m* = 0.23 from 4L to 3S). Various source–sink models as well as panmixia appeared less likely than this bidirectional steppingstone model. The outcome of this MIGRATE analysis supports the idea of well‐connected landward and seaward *Avicennia* populations along the Kidogoweni River, though dominated by an upstream movement reflecting flood tide rather than a downstream movement during ebb tide.

**TABLE 5:** Comparison of migration models on the directionality of gene flow between (a) seaward sites (S3, S5) and landward sites (L4, L6) of disjunct vegetation zones in close vicinity and along a same channel; and (b) seaward sites of three channels within Gazi Bay. The model with highest support is highlighted in gray. Connected populations with ←→ referring to bidirectional gene flow and → or ← to unidirectional gene flow

	Model	Directionality	Connected populations	Bezier log marginal likelihood	Model choice	Model probability
(a) Disjunct *Avicennia* zones						
	Panmixia	All	All	−752,872.35	4	0
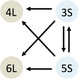	Source–sink	Unidirectional toward both landward sites	S3→L4 S3→L6 S5→L4 S5→L6 S3←→S5	−763,620.10	5	0
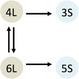	Source–sink	Unidirectional toward nearest seaward site	S3←L4 S5←L6 L4←→L6	−641,573.12	3	0
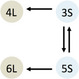	Source–sink	Unidirectional toward nearest landward site	S3→L4 S5→L4 S3←→S5	−618,439.78	2	0
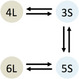	Steppingstone	Bidirectional	S3←→L4 S5←→L6 S3←→S5	−582,227.63	1	1
(b) Three channels of Gazi Bay						
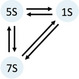	Panmixia	All	S1 + S5 + S7	−798,510.47	10	0
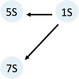	Source–sink	Unidirectional	S1 = Kinondo as source	−791,533.63	9	0
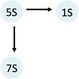	Source–sink	Unidirectional	S5 = Kidogoweni as source	−751,528.49	5	0
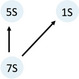	Source–sink	Unidirectional	S7 = Mkurumuji as source	−758,405.69	6	0
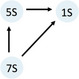	Source–sink	Unidirectional	S1 = Kinondo as sink	−786,194.54	8	0
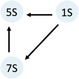	Source–sink	Unidirectional	S5 = Kidogoweni as sink	−713,559.57	4	0
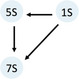	Source–sink	Unidirectional	S7 = Mkurumuji as sink	−685,826.31	3	0
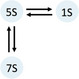	Steppingstone	Bidirectional	S1←→S5 S5←→S7	−781,722.11	7	0
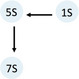	Steppingstone	Unidirectional	S1→S5→S7	−512,842.47	2	1
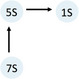	Steppingstone	Unidirectional	S1←S5←S7	−507,678.11	1	1

The connectivity among the mouth of three major water channels in Gazi Bay was best supported from a unidirectional steppingstone model (Table 5b). Both unidirectional models, either reflecting flood tide (7S→5S→1S) or ebb tide (1S→5S→7S), gave high likelihood values with nearly similar likelihood values, though each with a large difference to all other source–sink and panmixia models. The steppingstone model with a gene flow directionality reflecting tidal flow in the bay gave highest model probability. Estimates of mean population size for the populations of this best‐fit model were Theta = 0.25–0.42 with migration *M* = 9.2–9.4 (Bayesian analysis posterior distribution made available in Appendix S4). We obtained estimated values of *N* *e* *m* = 0.96 from the Mkurumuji channel (7S) to the Kidogoweni channel (5S) and

*N* *e* *m* = 0.91 from the Kidogoweni channel (5S) to the Kinondo channel (1S).

### 3.3 Fine‐scale genetic structure

**FIGURE 4:**
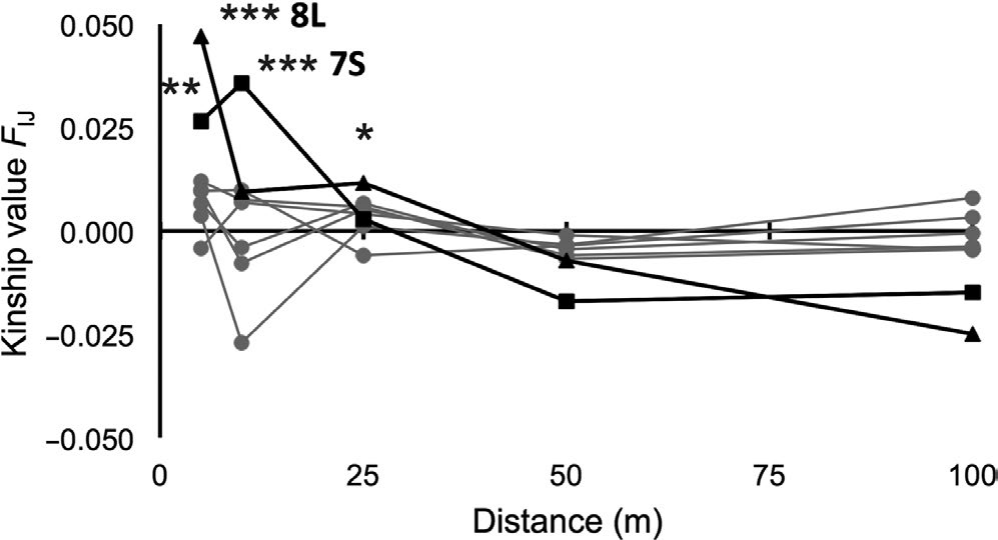
Fine‐scale spatial autocorrelation of *Avicennia marina* individuals showing kinship values (*F*_IJ_) over 10 m within seaward (7S) and up to maximum 25 m within landward (8L) transects of the narrow Mkurumuji channel (***p < .001; **p < .01; * *p* < .05). No structure occurred within transects of the wide Gazi Bay mangrove area (gray lines)

The spatial autocorrelation of individuals within transects of *A. marina* in Gazi Bay revealed an average intragroup kinship *F*_IJ_ = 0.061, however with an overall stronger kinship *F*_IJ_ = 0.072 at a mean distance of 5.6 m (*p* < .001) and *F*_IJ_ = 0.066 at a mean distance of 16.2 m (*p* < .001), with a log‐slope *b* = −0.008 (*p* < .001). A detailed analysis of the fine‐scale spatial genetic structure of each *A. marina* transect revealed only a significant different kinship value and slope within shortest distance class for sites of the southernmost channel, 7S and 8L (Figure 4). The kinship values were *F*_IJ_ = 0.026 (*p* = .008) and *F*_IJ_ = 0.036 (*p* = .001) for distance classes of 5 and 10 m, respectively, in transect 7S (Figure 4) and *F*_IJ_ = 0.047 (*p* = .001) and *F*_IJ_ = 0.012 (*p* = .018) for distance classes of 0–5 and 10–25 m, respectively, in transect 8L (Figure 4). The log‐slopes (distance) of the regression line were *b* = −0.02 (*p* = .001), similarly for both 7S and 8L. The overall *Sp* value for Gazi Bay was 0.009 but with a considerable range. The *Sp*‐statistic was low (*Sp* = 0.020–0.021) for sites 7S and 8L (Mkurumuji) that showed a fine‐scale spatial genetic structure and appeared even much lower (*Sp* = 0.0010–0.0080) for all other sites lacking a clear genetic structure at short distances. The estimated neighborhood size was limited to *Nb* = 47–49 in sites 7S and 8L, respectively, whereas elsewhere the estimates ranged from *Nb* = 125 to *Nb* = 982.

## 4 DISCUSSION

### 4.1 Landward–seaward sites

We found similar levels of genetic diversity for landward and seaward stands, which show no overall significant differentiation between both groups. Additionally, at an individual tree level, their low and nonsignificant kinship values suggest a mixed system, not dominated by a reciprocal flux between landward and seaward sites at close vicinity. More precisely, a bidirectional migration model revealed higher gene flow estimates from seaward to nearby landward sites than *vice versa*. This is consistent with findings from Dahdouh‐Guebas et al. (2004) in our study area, who reported less genetic exchange between *A. marina* stands from higher to lower intertidal zones, separated by zones that are dominated by trees from different mangrove species. Being hydrochoric, the transport of mangrove propagules depends on local water flow characteristics, which in an intertidal environment depends on the interaction of the local tidal regime with the landscape, and varies over the course of the tidal cycle. The dense aerial root system in the forest can strongly limit dispersal distances (Van der Stocken et al., 2015). However, while water currents may not always liberate propagules stuck deep into a dense mass of pneumatophores, retention by vegetation is expected to be rather low for the relatively small‐sized *A. marina* propagules, particularly at water levels that exceed the height of site‐specific root systems (Van der Stocken et al., 2015). Hence, low levels of landward–seaward patch connectivity are likely associated with differences in tidal energy (i.e., the asymmetry of tidal currents in the bay), water flow directionality, inundation period, and biological factors such as predation, which determine propagule availability and survival.

Tidal asymmetry in the area is characterized by weaker incoming flows than outgoing flows, promoting net export of matter from the mangrove system (Kitheka et al., 1997). This asymmetry in kinetic energy may result in an asymmetry of propagule deposition potential. Propagule deposition potential may be higher for propagules transported by the weaker flood tide, from seaward to landward stands, than for propagules transported by the stronger ebb currents, from landward to seaward stands. Propagule dispersal between stands also depends on water flow direction (Davis et al., 2017). Based on a release–recapture experiment near the 3S‐4L transect, Van der Stocken et al. (2015; see Figure 4 therein) reported two dominant dispersal directions (southward and west–northwestward) reflecting the site‐specific directionality of incoming and outgoing tides. Even though these experiments were focusing on *C. tagal* and *R. mucronata* propagules, results from these experiments suggest that transport along the 3S‐4L transect seems more likely from the seaward to landward site than *vice versa*, but seasonally dominant flows may offer other possibilities.

Landward and seaward zones are characterized by important differences in hydroperiod (duration of submergence). Differences in hydroperiod present different timeframes for dispersal *within* each zone, and temporally constrain the potential for gene flow *between* lower and higher intertidal sites. Low intertidal zones are flooded longer and more frequently, and experience greater water depths as well as stronger currents as compared to higher intertidal zones. As a result, the hydrokinetic energy needed to transport propagules will be available in seaward mangrove patches at moments when it is absent in more landward zones. Short hydroperiods are particularly the case for the sites 2L and 6L, which inundate only near spring tide (T. Van der Stocken, personal observation). Hence, only during a limited time frame of the monthly tidal cycle are landward sites hydrologically connected to the more seaward stands, and can propagules be transported directly between mangrove patches at different elevations in the intertidal zone. Importantly, these differences in hydroperiod also present different windows of opportunity for propagules to strand and develop roots (Balke et al., 2011). Propagules that are transported from lower to higher intertidal zones and subsequently strand in the higher intertidal (landward) zone will experience longer inundation‐free periods (promoting establishment) compared to propagules from landward stands that strand in more often flooded seaward zones. Even though the chance of retention by vegetation is lower in the less densely grown higher intertidal *A. marina* stands, consisting of open canopy shrub forest, the tidal energy in the higher intertidal is low (N. Koedam, personal observation), leaving a large amount of litter in a relatively long inundation‐free time window.

Increased potential for establishment in landward patches is also favored by the lower hydrodynamic forces from waves and currents, compared to landward zones. In a study on the mangrove forest structure dynamics in Gazi Bay, Di Nitto et al. (2008) noted that within a time span of 4 days, propagules could be washed away by the flood tide. The authors reported that this was particularly the case at the seaward side where hydrokinetic energy from waves is higher than in landward stands. Along its way through the mangrove forest, vegetative structures such as stems, roots, and leaves can strongly reduce wave and current energy (Mazda et al., 2006; Vanegas et al., 2019) that may otherwise obstruct propagule establishment (Balke et al., 2011).

Another explanation for these results could be related to different predation rates in landward and seaward sites. In our study area, for example, grapsid crabs (particularly *Neosarmatium africanum*) were shown to clear nearly 100% of the propagules in landward stands (Dahdouh‐Guebas et al., 1997, 1998) with fast consumption of *A. marina* propagules compared to propagules from other mangrove species, and this particularly under *A. marina* canopy (Van Nedervelde et al., 2015). Hence, despite the large number of propagules produced in *A. marina* (Clarke, 1992), high predation rates in landward stands in our study area may strongly reduce the number of potential migrants from landward to seaward zones. Overall, these asymmetries in tidal currents (strength and directionality), hydroperiod, and predation rates, are consistent with our MIGRATE results, indicating higher seaward‐to‐landward than landward‐to‐seaward migration. A further explanation could be a possible effect on directionality of gene flow caused by pollinator movements (Hermansen, et al., 2014). Pollen flow could not be tested from our data on adult trees, as such analysis would require a different design including mother trees and their propagules. However, considering present results, we assume the effect of pollen flow to be of minor importance because connectivity between nearby landward sites was not supported by any MIGRATE model. Pollination over rather short distances has also been suggested by Hermansen, et al. (2014). In their study in the Sydney region (Australia), Hermansen, et al. (2014) found that within large and small *A. marina* stands pollen grains are typically dispersed within individual trees or between a limited number of directly adjacent trees.

### 4.2 Channel network structure

Instead of a consistent disjunct landward–seaward zonation, spatial genetic patterns within Gazi Bay reflect the local channel network architecture. Our genetic analyses (*F*_ST_, PCoA, STRUCTURE, and BARRIER) revealed significant genetic differentiation between sites that are situated along different water channels (Kinondo, Kidogoweni, and Mkurumuji). High longitudinal (i.e., along‐channel) connectivity was found for the Kidogoweni channel but not for Mkurumuji (it was not tested along the Kinondo channel, where we considered only one location). Overall, this is consistent with findings that stream channels may act as corridors for dispersal (Johansson et al., 1996; Schmiedel & Tackenberg, 2013) and that connectivity in water‐dispersed species is typically low between sites that are not well connected hydrologically (Hughes et al., 2009). The absence of high connectivity along the Mkurumuji most likely reflects the lower influence of tides in this channel compared to Kidogoweni, due to the channel's orientation perpendicular to the directionality of tidal currents. In contrast, strong semi‐diurnal tides entering the bay from the south (Kruyt & van den Berg, 1993) may greatly influence dispersal dynamics along the Kidogoweni channel that is oriented predominantly north–south.

The role of river network structure in shaping the genetic variation within and between populations has been commonly investigated for a broad range of freshwater organisms, including fish (Shao et al., 2019; Thomaz et al., 2016), insects (Finn et al., 2006, 2007), and plants (Sander et al., 2018). In these studies, the observed population genetic patterns are generally described using four connectivity models that predict how populations with different life history traits and dispersal capabilities interact within their structured riverine habitat (Finn et al., 2007; Hughes et al., 2009). Generally, the explanatory power of the river network is expected to be stronger for species with no or limited capacity for terrestrial (among‐stream) movement and short floating abilities, and in riverine systems with permanent downstream and confined (unidirectional) water flow (Tonkin et al., 2017). As in riverine networks, dispersal traits, and the physical and hydrodynamic characteristics of the mangrove landscape are important factors regulating dispersal (Van der Stocken et al., 2019). However, in contrast to riverine systems where the transport of passive propagules is predominantly downstream (Tonkin et al., 2017), coastal processes such as tides and waves add to the complexity of dispersal in these coastal settings (Di Nitto et al., 2008). For example, in the Normanby River estuary (Northeast Australia), Stieglitz and Ridd (2001) found that mangrove propagules were moving upstream from the mangrove fringe, trapped in an axial convergence generated by a density‐driven circulation cell. Using genetic data and release–recapture experiments, Ngeve et al. (2017) found bidirectional propagule flow along the Wouri River in Cameroon, reflecting the interaction of river flow and tides. Indeed, in the lower reaches of estuaries, tidal discharge may greatly exceed river discharge (Pritchard, 1956), and drive gene flow upstream during flood tide and downstream during ebb tide. This tidal effect likely explains the overall isolation by distance within the bay, the absence of strong genetic differentiation between mangroves along the same channel, as well as the absence of greater genetic diversity downstream of confluences as predicted in systems dominated by riverine regimes (Thomaz et al., 2016).

The outcome of our MIGRATE analysis supports the hypothesis of channels being connected in a stepwise manner instead of an extensive mixing regime throughout the entire bay. Both unidirectional steppingstone models correspond to the dominant directionality of flood and ebb tide acting through the 3.5‐km‐wide entrance in the southern part of the bay, slightly more reflecting tidal flood than ebb. This pattern may potentially be explained by the slower flood tide that may transport propagules gradually upstream *within* channels, while the stronger ebb tide may result in lower chances of deposition/retention and hence establishment. The younger and diverse stand near the mouth of the Mkurumuji (7S) was not a subset of the older mangrove along Kidogoweni (5S), supporting the abovementioned flood‐mediated directionality from south to north. Significant genetic differentiation *among* channels may result from the tidal asymmetry in the region, characterized by stronger ebb than flood tide and resulting in a net export (Kitheka et al., 1996). This net transport out of the bay may reduce the chance of propagules from one channel being transported out of this channel and then upstream into another channel. Direct among‐channel transport has been observed in Amazonian studies, where channel overflow during rainy seasons may facilitate long‐distance propagule transport (De Campos et al., 2013). However, while fluctuations in water level over the course of the tidal cycle may allow for regular channel overflow in our study site, direct lateral connectivity between the Kinondo and Kidogoweni channels may occur only during times of elevated tidal height or extreme events. In the three different channels, the potential for among‐channel connectivity decreases for sites that are located more upstream as the physical distance between the channels increases (Tonkin et al., 2017). In a way, this is illustrated also by the shape of the mangrove forest's inland fringe, which fans out along the channel network and diverges toward the channel heads (Figure 1).

### 4.3 *Fine‐scale genetic structure within* Avicennia *stands*

The overall *Sp* value (ca. 0.009) found for *A. marina* in Gazi Bay is among the ranges reported for outcrossing trees in general (Vekemans & Hardy, 2004). Remarkably, *Sp* values for *A. marina* along the Mkurumuji (ca. 0.02) come close to patterns for “gravity‐dispersal” (mean *Sp* = ca. 0.028; see Table 3 in Vekemans & Hardy, 2004) referring to local conditions of retention, whereas results for all other sites suggest open systems with ample dispersal, certainly beyond neighboring trees (on average further away than 21 m). The obtained *Sp* values for *A. marina* in Gazi Bay are comparable to *Sp* values previously reported for *A. germinans* populations from estuaries in Northwestern Mexico (Millán‐Aquilar et al., 2016), which ranged from 0.002 to 0.015 in adult trees, and could increase for saplings up to 0.035. An overall estimate of *Sp* = 0.0186 was obtained for the same species in Caribbean and Pacific estuaries of Panama (Céron‐Souza et al., 2012). It must be noted that the first distance class considered in each of these studies is very different: Where the first distance class in our study includes all pairs of individuals within 5 m distance (in order to capture the fine‐scale spatial genetic structure for *A. marina*), the abovementioned studies on *A. germinans* considered first distance classes of 0–50 m (Millán‐Aquilar et al., 2016) and 0–100 m (Céron‐Souza et al., 2012), which might not capture the full spatial genetic structure. Remarkably, despite such a tenfold difference in minimal distance of the sampling design, the range of *Sp* values for *Avicennia* is comparable. This can be explained from the lower kinship values but stronger slope of the relationship between kinship and log distance in Gazi Bay compared to those in both studies on *A. germinans*. For mangrove sites located along the same channel, admixed gene pools without any fine‐scale structure were found for Kidogoweni, suggesting open systems within these transects < 100 m. It can be hypothesized that extensive pollinator movements within a transect (Hermansen, et al., 2014) may reduce or even nullify its fine‐scale structure. On the contrary, elevated kinship values and fine‐scale structure were detected within a distance class of 5 to 20 m, only in the two Mkurumuji sites (7S and 8L). Presence or absence of fine‐scale spatial genetic structure is most likely due to differences in the relative orientation of both channels with regards to the direction of tidal currents. Kidogoweni is positioned near‐parallel to the direction of tidal currents. Hence, the mangrove patches along this channel are much more exposed to the tidal currents than patches along the Mkurumuji channel, which is oriented more or less perpendicular to the tidal currents entering the bay in the south. Even though freshwater discharge can be high for Mkurumuji during the wet season, the riverine energy flux is confined predominantly within the channel with limited overflow, reducing the chance of propagules from 8L to be exported to open waters.

## 5 CONCLUSION

As a conclusion, the genetic diversity levels were comparable between seaward and landward *A. marina* mangrove patches and revealed no overall genetic differentiation between these spatially disjunct zones. Gene flow appears to be governed by incoming tides from seaward to nearby landward sites, perpendicular to a channel. The genetic structure of *A. marina* within the bay corresponds to the channel network structure, and channel connectivity was most supported by unidirectional steppingstone models corresponding to the dominant directionality of flood and ebb tide. A fine‐scale spatial genetic structure was absent for mangrove patches located along the north‐south oriented and wide Kidogoweni channel, but was clearly present along a less tidally influenced channel. Overall, our findings show that patterns of *A. marina* connectivity are explained by hydrological proximity, channel network structure, and hydrokinetic energy, rather than just their positioning as disjunct landward or seaward zones.

## CONFLICT OF INTEREST

The authors declare no conflicts of interest.

## AUTHOR CONTRIBUTION

**Ludwig Triest:** Conceptualization (lead); Data curation (lead); Formal analysis (lead); Funding acquisition (equal); Investigation (lead); Methodology (lead); Resources (lead); Visualization (supporting); Writing‐original draft (lead); Writing‐review & editing (supporting). **Tom Van der Stocken:** Conceptualization (lead); Investigation (lead); Methodology (lead); Visualization (lead); Writing‐original draft (lead); Writing‐review & editing (lead). **Abbie Allela Akinyi:** Data curation (supporting); Formal analysis (supporting). **Tim Sierens:** Formal analysis (supporting); Methodology (supporting). **James Kairo:** Data curation (supporting); Funding acquisition (equal). **Nico Koedam:** Conceptualization (lead); Funding acquisition (equal); Investigation (supporting); Project administration (lead); Resources (lead); Writing‐review & editing (supporting).

## Supporting information

Appendi S1‐S4

## Data Availability

Sequence data can be found in NCBI GenBank (https://www.ncbi.nlm.nih.gov/genbank/), AMK microsatellites with accession numbers MT713342–MT713346. Microsatellite data of *Avicennia marina* from Gazi Bay (Kenya) are available at Dryad (https://doi.org/10.5061/dryad.v9s4mw6t2).
