# Supplementary material for: Channel network structure determines genetic connectivity of landward–seaward Avicennia marina populations in a tropical bay
Source: Ecol Evol. 2020 Oct 16;10(21):12059–75. doi: 10.1002/ece3.6829 (PMC7663977; doi:10.1002/ece3.6829)

**Appendix 1.** Features of 10 primers (4 newly developed) and microsatellite loci for *Avicennia marina*. Total number of alleles (A), Allelic richness (*A_R_* at k = 46 diploid individuals), overall observed heterozygosity (*H*o) and overall expected heterozygosity (*H*e), within population inbreeding (*F*_IS_) and among population differentiation (*F*_ST_) are given for 8 sites in Gazi Bay (Average N = 55 to 57).

| Locus | Primer sequence (5' - 3') | Repeat motif | Size | A | *A_R_* | *Ho* | *He* | *F*_IS_ | *F_ST_* | GenBank  accession no. |
| --- | --- | --- | --- | --- | --- | --- | --- | --- | --- | --- |
| Avma01* | F: TCTCTCTCTCTCACACACACAC | (TC)6(AC)8 | 128-130 | 2 | 2 | 0.300 | 0.346 | 0.129 | 0.051 | EF462475 |
|  | R: VIC-CCAAAGAGTCACAGCAGAGGCACT |  |  |  |  |  |  |  |  |  |
| Avma02* | F: TCTCTCTCTCTCACACACACAC | (TC)6(AC)9 | 86-101 | 7 | 6.2 | 0.660 | 0.771 | 0.151 | 0.043 | EF462474 |
|  | R: VIC-CTGTGTTGAGGTGGTTGATGAGAT |  |  |  |  |  |  |  |  |  |
| Avma08* | F: TCTCTCTCTCTCTCACACAC | (TC)6(AC)7(AG)2 | 173-183 | 6 | 4.3 | 0.633 | 0.590 | -0.061 | 0.081 | EF462478 |
|  | R: 6FAM-GGGCGAGGAGATGGGAAATTAG |  |  |  |  |  |  |  |  |  |
| Avma10* | F: TCTCTCTCTCTCTCACACAC | (TC)6(AC)10 | 73-80 | 5 | 3.6 | 0.560 | 0.512 | -0.058 | 0.055 | EF462480 |
|  | R: NED-CACCATTATATCTAGTGGCTTGTG |  |  |  |  |  |  |  |  |  |
| Avmal7* | F: TCTCTCTCTCTCTCACACAC | (TC)6(AC)10 | 70-76 | 4 | 3.4 | 0.435 | 0.450 | 0.035 | 0.028 | EF462482 |
|  | R: PET-GCACTACCTGTTGATAGAGC |  |  |  |  |  |  |  |  |  |
| M3° | F: PET-GGTTCCTGCAAGTATGTCAACACCCTC | (TG)15 | 181-191 | 6 | 5.2 | 0.538 | 0.545 | 0.017 | 0.069 | No number |
|  | R: ACCTCGATTCCTCCCCGAATGC |  |  |  |  |  |  |  |  |  |
| AMK6§ | F: 6FAM-CCCCTGTAGCTTCTGATTTAG | (GA)13 | 121-123 | 2 | 2 | 0.182 | 0.175 | -0.030 | 0.253 | MT713342 |
|  | R: GACCAAGACTCGTTAAATTCC |  |  |  |  |  |  |  |  |  |
| AMK10§ | F: 6FAM-TTCACGAAGCTATAATTTTCC | (CT)19 | 136-158 | 9 | 5.9 | 0.502 | 0.518 | 0.036 | 0.045 | MT713343 |
|  | R: GGGGCTATTCGATTAACACTA |  |  |  |  |  |  |  |  |  |
| AMK18§ | F: VIC-AATTCAGCATCAACAGACAAG | (AC)13 | 211-219 | 4 | 2.9 | 0.415 | 0.433 | 0.040 | 0.082 | MT713344 |
|  | R: AGGAAGAATAATGTTCCCTATTT |  |  |  |  |  |  |  |  |  |
| AMK34§ | F: NED-TCGCATCATAAGAAACCTACT | (CT)14 | 117-135 | 7 | 6.7 | 0.569 | 0.619 | 0.088 | 0.034 | MT713346 |
|  | R: TGGACTAAACTTTAAGCGAAA |  |  |  |  |  |  |  |  |  |

Primers from *(Geng et al., 2007), °(Maguire et al., 2000b). § are newly developed primers for this study from source material of Gazi Bay.

**Appendix 2.** Probability of identity (*PI*) for an increased combination of loci in each population and when considering putative siblings.


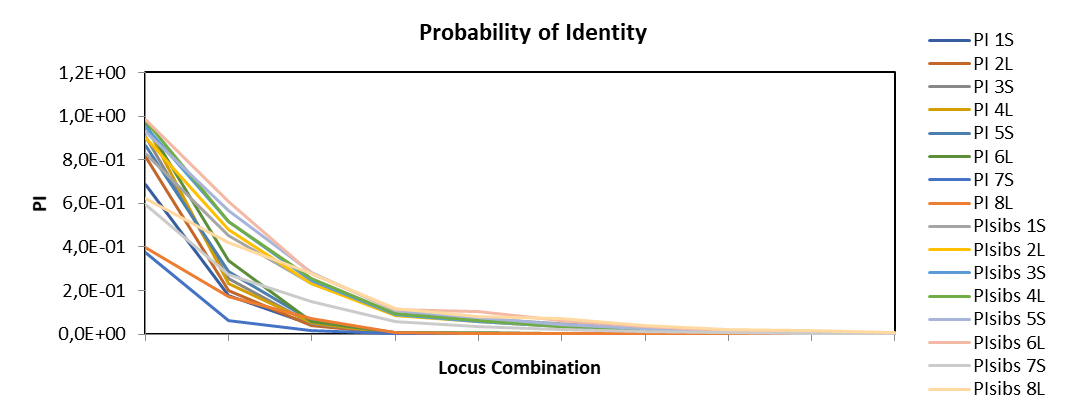


**Appendix 3.** (a) PCoA of 475 *Avicennia marina* individual genotypic differences; (b) PCoA of eight transects in Gazi Bay.


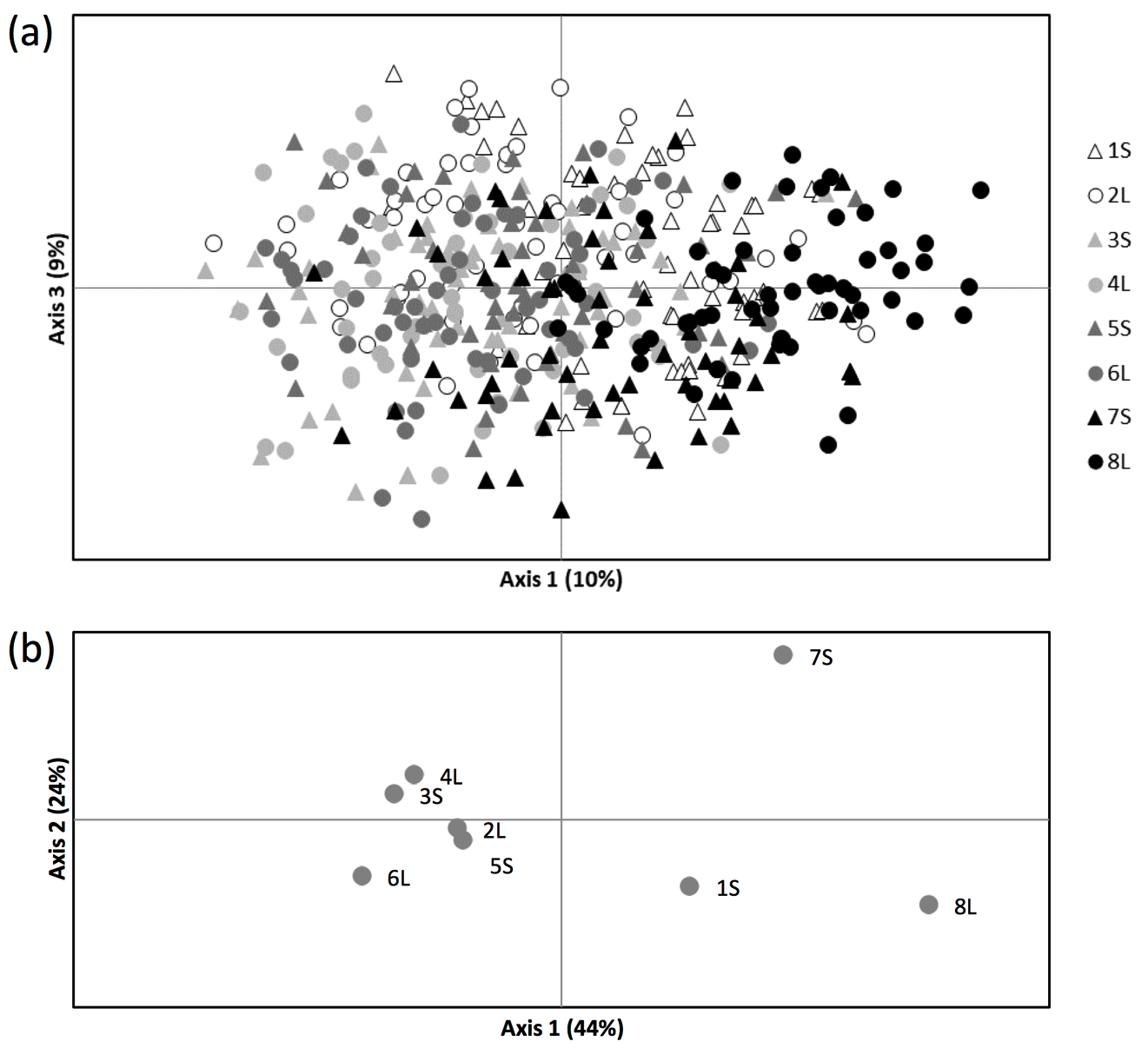


**Appendix 4.** Bayesian posterior probability distribution tables and corresponding histograms (Freq) over all loci for the theta (θ) and migration/gene flow (M) values of landward-seaward populations along the Kidogoweni channel (S3, L4, S5 and L6; scenario A), and between seaward populations at the mouth of the three major channels in Gazi Bay (S1, S5 and S7; scenario B). The selection of the different scenarios are based on the Bayesian analysis best-fit models as obtained with MIGRATE software. The color code in the histograms corresponds to estimates within the different credibility intervals and red lines are the prior probability distribution.

| **Scenario A (Landward – Seaward) Sites S3 L4 S5 L6** | | | | | | | |
| --- | --- | --- | --- | --- | --- | --- | --- |
| *Stepping stone bidirectional (model choice 1)* | | | | | | | |
| Population | S3 | L4 | S5 | L6 |  |  |  |
| S3 | * | * | * | 0 |  |  |  |
| L4 | * | * | 0 | 0 |  |  |  |
| S5 | * | 0 | * | * |  |  |  |
| L6 | 0 | 0 | * | * |  |  |  |
| Loci Parameter | 2.5% | 25.0% | Mode | 75.0% | 97.5% | Median | Mean |
| All Theta_1 | 0.22667 | 0.33333 | 0.40667 | 0.45333 | 0.52000 | 0.40667 | 0.39101 |
| All Theta_2 | 0.14667 | 0.28000 | 0.36667 | 0.42667 | 0.50667 | 0.36667 | 0.34417 |
| All Theta_3 | 0.21333 | 0.33333 | 0.40667 | 0.45333 | 0.52000 | 0.40667 | 0.38623 |
| All Theta_4 | 0.02667 | 0.12000 | 0.16667 | 0.17333 | 0.17333 | 0.16667 | 0.16772 |
| All M_2 🡪 1 | 0.28000 | 0.28000 | 0.43333 | 0.44000 | 0.44000 | 2.42000 | 2.38099 |
| All M_3 🡪 1 | 1.25333 | 2.54667 | 3.10000 | 3.68000 | 5.00000 | 3.16667 | 3.22305 |
| All M_1 🡪 2 | 8.36000 | 9.25333 | 9.54000 | 9.69333 | 9.85333 | 9.36667 | 9.24287 |
| All M_1 🡪 3 | 6.80000 | 8.38667 | 9.52667 | 9.69333 | 9.82667 | 7.88667 | 6.65715 |
| All M_4 🡪 3 | 0.00000 | 0.00000 | 0.27333 | 0.28000 | 0.28000 | 1.07333 | 1.06811 |
| All M_3 🡪 4 | 8.40000 | 9.20000 | 9.50000 | 9.61333 | 9.82667 | 9.32667 | 9.23556 |


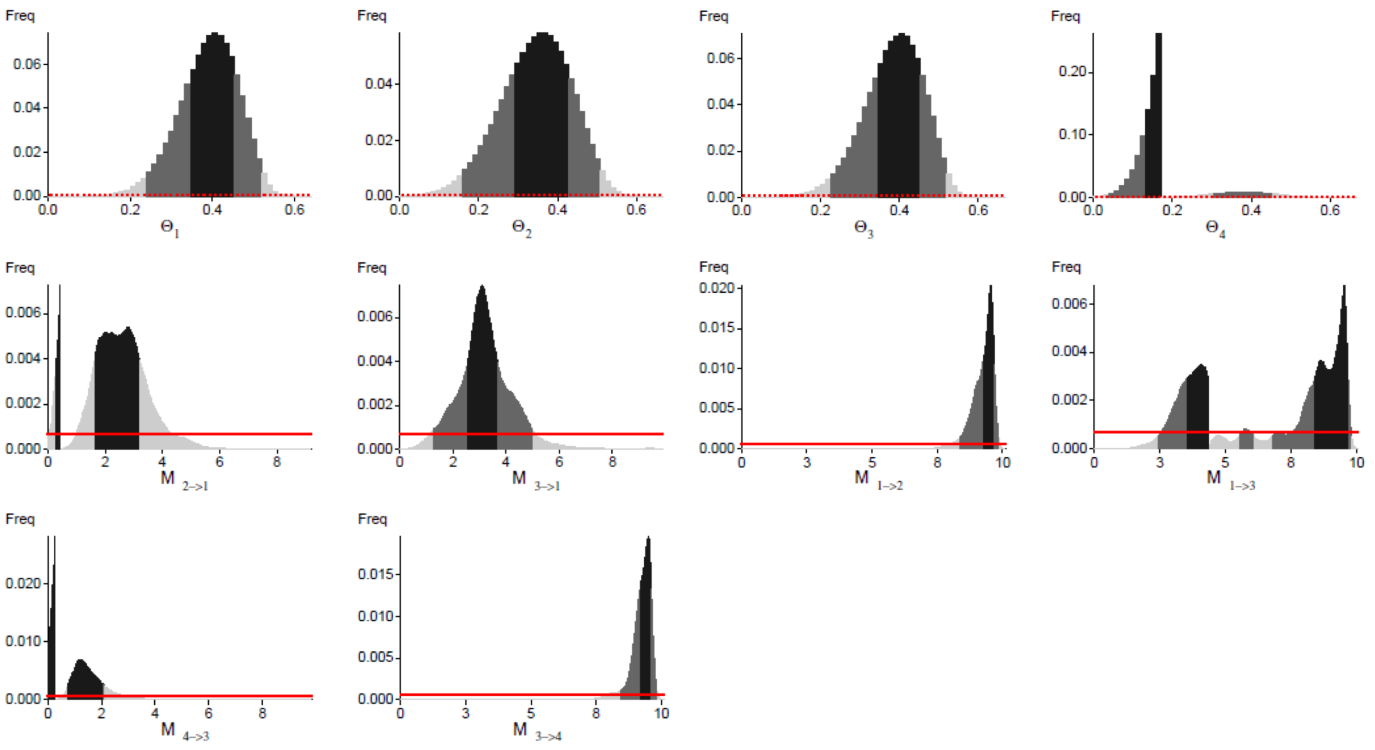


| **Scenario B (Seaward between three channels) Sites 1S 5S 7S** | | | | | | | |
| --- | --- | --- | --- | --- | --- | --- | --- |
| *Steppingstone unidirectional (model choice 1)* | | | | | | | |
| Population | S1 | S5 | S7 |  |  |  |  |
| S1 | * | * | 0 |  |  |  |  |
| S5 | 0 | * | * |  |  |  |  |
| S7 | 0 | 0 | * |  |  |  |  |
| Loci Parameter | 2.5% | 25.0% | Mode | 75.0% | 97.5% | Median | Mean |
| All Theta_1 | 0.16000 | 0.28000 | 0.35333 | 0.41333 | 0.50667 | 0.36667 | 0.34319 |
| All Theta_2 | 0.25333 | 0.36000 | 0.43333 | 0.48000 | 0.54667 | 0.43333 | 0.41556 |
| All Theta_3 | 0.10667 | 0.20000 | 0.24667 | 0.25333 | 0.25333 | 0.24667 | 0.24735 |
| All M_2 🡪 1 | 8.62667 | 9.36000 | 9.54000 | 9.66667 | 9.86667 | 9.46000 | 9.36127 |
| All M_3 🡪 2 | 8.32000 | 9.25333 | 9.52667 | 9.62667 | 9.81333 | 9.28667 | 9.17997 |


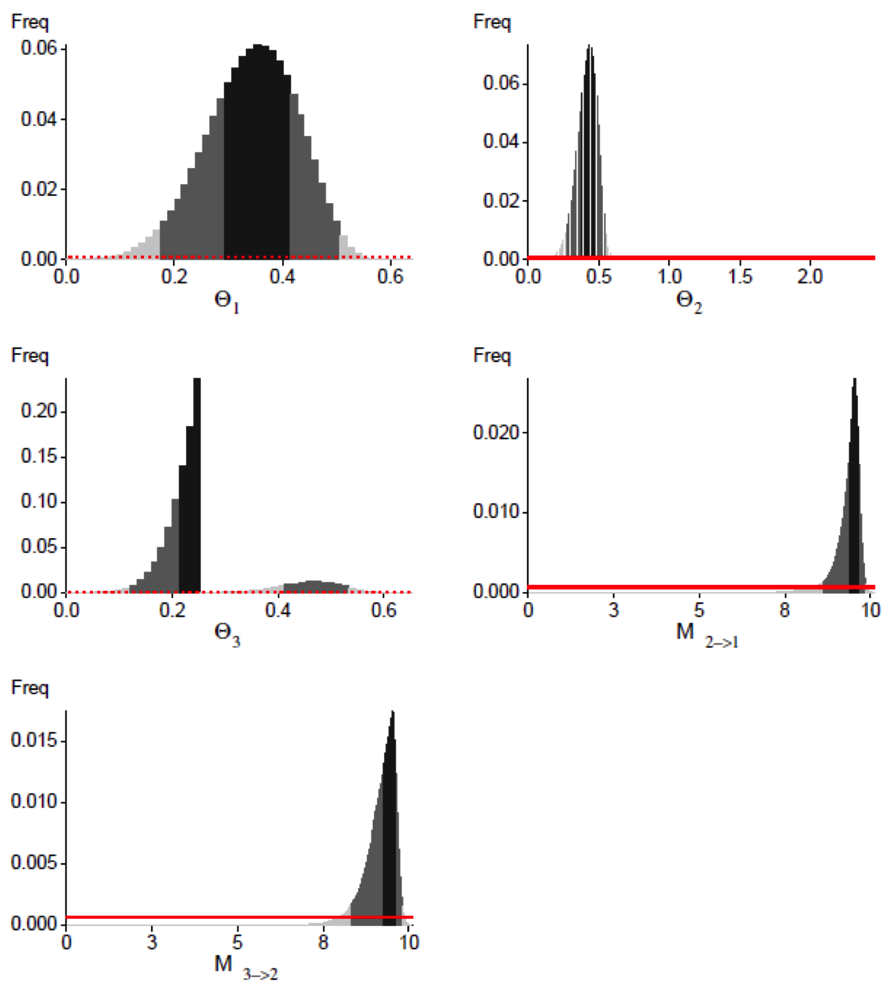

Supplement: Supplementary file 1 — Appendi S1‐S4 [file ECE3-10-12059-s001.docx]
